# Assessing essential oils from Macaronesia: a study on their efficacy against phytopathogenic and obligate biotrophic fungi

**DOI:** 10.1002/ps.70205

**Published:** 2025-09-18

**Authors:** Rui Miguel Ferreira, Verónica Sousa Pereira, Duarte Fernandes Sardinha, Samuel Rodríguez Sabina, Raimundo Cabrera Pérez, Paula Machado Castilho

**Affiliations:** ^1^ CQM – Centro de Química da Madeira Universidade da Madeira Funchal Portugal; ^2^ Laboratório de Qualidade Agrícola, Direção de Serviços dos Laboratórios Agrícolas e Agroalimentares, Direção Regional de Agricultura e Desenvolvimento Rural, Secretaria Regional de Agricultura, Pescas e Ambiente Camacha Portugal; ^3^ Unidad de Fitopatologia, Sección de Biologia Universidad de La Laguna Tenerife Spain

**Keywords:** essential oils, phytopathogenic fungi, obligate fungi, antifungal activity

## Abstract

**BACKGROUND:**

Essential oils (EOs) extracted from eight culinary herbs and spices, some endemic to Macaronesia flora (*Cedronella canariensis, Clinopodium ascendens* and *Laurus novocanariensis*) and others common in Mediterranean cuisine (*Cinnamomum burmannii*, *Ocimum gratissimum*, *Origanum vulgare* subsp. *virens*, *Syzygium aromaticum* and *Thymus vulgaris*) were evaluated via direct‐contact bioassays, both *in vitro* and *in vivo*, against three species of phytopathogenic fungi (*Alternaria alternata, Botrytis cinerea*, and *Fusarium oxysporum*) and the obligatory biotrophic fungus *Oidium farinosum*. Carvacrol‐rich *Origanum vulgare* was selected to study the influence of isomerism on bioactivity, because *Thymus vulgaris* is rich in thymol, a structural isomer of carvacrol.

**RESULTS:**

Five EOs exhibited strong to moderate antifungal activity and were further screened at lower concentrations to assess their toxicity and growth inhibition thresholds. *Cedronella canariensis* showed no antifungal activity, whereas *Laurus novocanariensis* demonstrated only weak activity against *Botrytis cinerea*. This fungus was more susceptible to the tested EO than the other phytopathogenic fungi. *Origanum vulgare* was slightly more effective than *Thymus vulgaris*, for all three phytopathogenic fungi. *Ocimum gratissimum*, *Syzygium aromaticum*, and *Cinnamomum burmannii* significantly reduced powdery mildew severity, whereas *Origanum vulgare* and *Thymus vulgaris* showed moderate activity.

**CONCLUSION:**

Phenylpropanoid‐rich EOs, such as *Cinnamomum burmannii, Ocimum gratissimum*, and *S. aromaticum*, exhibited the strongest antifungal activity in both bioassays with phytopathogenic and obligate biotrophic fungi. Our results confirm the higher antifungal activity of the phenylpropanoid class of EOs. © 2025 The Author(s). *Pest Management Science* published by John Wiley & Sons Ltd on behalf of Society of Chemical Industry.

## Graphical abstract

- Essential oils (EOs) extracted from eight culinary herbs and spices were evaluated via direct‐contact bioassays, both *in vitro* and *in vivo*, against three species of phytopathogenic fungi and one obligatory biotrophic fungus.
- Phenylpropanoid‐rich EOs presented the strongest antifungal activity in both bioassays with the phytopathogenic and the obligate biotrophic fungus.

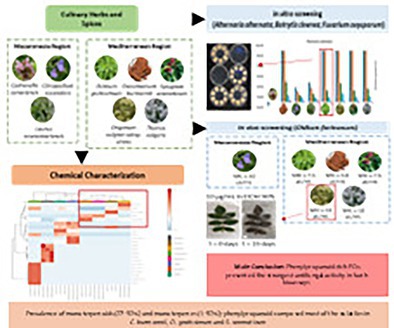

## 1 INTRODUCTION

Macaronesia is a region composed of the Azores, Madeira, Canary Islands, and Cape Verde archipelagos, in which the agricultural sector plays a significant role in the local economy. These archipelagos have diverse climatic conditions and are ideal for the cultivation of numerous tropical, subtropical, and temperate crops, such as tomatoes, potatoes, avocados, annonas, bananas, grapes, and mangoes.^1^ Most of these islands are small with a high population density and crops tend to grow remarkably close to each other. Cultivated plants are susceptible to pre‐harvest diseases induced by phytopathogenic fungi, which contribute to diminished crop yield and economic losses. For example, *Botrytis cinerea* Pers. is responsible for pre‐ and postharvest diseases by causing spots, blights, and gray mold in the soft tissues of a wide variety of crops, including grapes and ornamental flowers.^2^, ^3^, ^4^ *Fusarium oxysporum* Schltdl. is the fifth most economically important plant pathogen and causes vascular wilt in several host plants, including tomato, banana, and melon.^5^ *Alternaria alternata* (Fr.) Keissl. is another destructive fungus known to induce leaf spots and blight in soybean plants and postharvest diseases during the storage and transportation of vegetables and fruits, such as potatoes, pears, and tomatoes.^6^, ^7^

Cultures can also be affected by powdery mildew, an obligate biotrophic fungus with more than 10 000 plant hosts, ranging from cereals and fruits to ornamental flowers.^8^, ^9^ Its infection is characterized by the presence of powdery white spots, which can be identified in individual plant tissues and cause significant productivity losses worldwide, despite the need for their plant host to survive to obtain nutrients and complete their life cycle.^8^, ^9^ For instance, the apple powdery mildew induced by *Podosphaera leucotricha* (Ellis & Everh.) E.S. Salmon, also known as *Oidium farinosum* Cooke, can infect blossoms and inhibit fruit production or lead to fruits with small, stunted, and/or russet features.^10^ The Canary archipelago region is the main European producer of papaya, a tropical fruit highly susceptible to several species of powdery mildew fungi.^11^ The current use of site‐specific fungicides has led to the development of fungicide resistance in powdery mildew. This resistance is attributed to the plastic genomes of these fungi, which facilitate their rapid and devastating spread worldwide.^8^, ^9^

Pesticide dependence in the Macaronesia region is high, resulting in the accumulation of chemical residues that degrade and contaminate the soil.^1^ These observations also reflect the global problem surrounding the intense overuse of pesticides, and the search for eco‐friendly and efficient pesticides has increased in recent years. Botanical pesticides have attracted attention as potential natural pesticides because they include secondary metabolites produced by plants for protection against biotic and abiotic factors.^12^ The Macaronesia region has unique rainforests that can be potential sources of botanical pesticides. The insecticidal activity of ethanolic extracts from seven plants of Macaronesia against the fly *Ceratitis capitata* Wiedemann was recently studied.^13^ However, little is known about the biopesticide potential of essential oils (EOs) derived from Macaronesia plants.

This study aimed to evaluate the fungicidal activity of eight EOs from plants used as culinary herbs and spices on Madeira Island (both endemic and introduced) against phytopathogenic fungi (*B. cinerea*, *F. oxysporum*, and *A. alternata*) and powdery mildew (*Oidium farinosum*). For this purpose, the chemical compositions of the obtained EOs were analyzed using gas chromatography–flame ionization detector (GC–FID), and the chemical compositions of the obtained EOs were compared with those of standard and commercialized EOs. The EO compositions of some of these plants have been analyzed previously, and the results published^14^, ^15^; however, new collections and analyses were performed for this study, with new data that emphasize the seasonal variability of these oils. The antifungal activity was evaluated using *in vitro* and *in vivo* bioassays, with the EOs screened at lower concentrations to determine the toxicity and growth inhibition thresholds.

## 2 MATERIALS AND METHODS

### 2.1 Plant material

Fresh leaves of distinct species relevant to the Macaronesia flora [*Cedronella canariensis* (L.) Webb & Berthel, *Clinopodium ascendens* (Jord.) Samp., and *Laurus novocanariensis* Rivas Mart., Lousã, Fern. Prieto, E. Días, J.C. Costa & C. Aguiar] were collected from various locations on Madeira Island. As described in Table 1, vouchers for endemic species were deposited in the Madeira Botanical Garden *‘Eng. Rui Vieira*’. Aromatic herbs and spices [*Cinnamomum burmannii* (Nees & T. Nees) Blume, *Origanum vulgare* subsp. virens (Hoffmanns & Link) Bonnier & Layens, *Syzygium aromaticum* (L.) Merr. & L.M. Perry, and *Thymus vulgaris* L.] were acquired from a specialized market. *Ocimum gratissimum* was sourced from a private collection, cultivated in substrate, and subjected to controlled irrigation and foliar fertilizing treatment, specifically for this work. The total aerial parts were dried for 48 h in a ventilated food dehydrator at 40 °C. After drying and evaluation of the humidity content using a Kern DBS humidity scale (Kern‐Sohn, Ballinger, Germany), the material was ground to a fine powder in a mechanical grinder to obtain particles of size <250 μm.

**Table 1:** Plant species ID, origin, development phase and seasonal period of collection and essential oil extraction yield

Plant species	Plant development phase	Origin	Seasonal period of collection	ID voucher	Yield (v/w, %)
*Cedronella canariensis*	Vegetative	Fajã da Nogueira – Madeira	Spring 2021	MADJ24356	1.10
*Cinnamomum burmannii*	n/a^†^	Market Funchal – Madeira	n/a^†^	n/a^†^	0.62
*Clinopodium ascendens*	Vegetative	Fajã da Nogueira – Madeira	Spring 2021	MADJ306406	1.42
*Laurus novocanariensis*	Vegetative	Caniço‐Madeira	Fall 2021	MADJ41268	0.36
*Ocimum gratissimum*	Flowering	Jardim das Aromáticas – Madeira	n/a^†^	n/a^‡^	0.30
*Origanum vulgare* subsp. *virens*	Flowering	Parque Ecológico do Funchal – Madeira	Spring 2021	MADJ306206	1.90
*Syzygium aromaticum*	n/a^†^	Market Funchal – Madeira	n/a^†^	n/a^†^	1.00
*Thymus vulgaris*	n/a^†^	San Cristóbal de La Laguna – Tenerife, Canary Island	n/a^†^	n/a^†^	0.37

^†^ Commercially acquired.

^‡^ Cultivated in substrate and subject to controlled irrigation and foliar fertilizing treatment.

n/a, not applicable.

### 2.2 EO extraction

For each sample, the EOs were extracted by hydrodistillation for 4 h at a ratio of 1:20 dry plant/water (w/v) using a Clevenger‐type apparatus. The EOs were collected, dried over sodium sulfate, and stored in an opaque flask at 4 °C. The extraction yields for each sample are presented in Table 1.

### 2.3 Phytochemical analysis

#### 2.3.1 Chemicals and standards

Standards were used for the identification of phytochemical analysis by GC–FID: (−) pulegone (98%), (−) isopulegone (99%), (+) *p*‐menth‐1‐ene (98%), (−) citronellal (98%), (−)‐*β*‐caryophyllene (98%), *ƴ*‐terpinene (99%), *α*‐terpinene (95%), *p*‐cymene (99%), (−)‐*α*‐pinene (98%), (−)‐*β*‐pinene (99%), *R* (+) limonene (97%), and eugenol (99%) were acquired from Fluka (Buchs, Switzerland). Thymol (99%), carvacrol (98%), and carvacrol methyl ether (98%) were acquired from Merck KGaA (Darmstadt, Germany). *n*‐Hexane (95%) was acquired from PanReac AppliChem (Catellar del Valles, Spain). *Trans*‐cinnamaldehyde (≥ 98%) was acquired from Thermo Fisher Scientific (Darmstadt, Germany), and (−)‐menthol was acquired from TCI Chemicals (Tokyo, Japan). For fungicide/fungistatic activity, potato glucose agar (PGA) and tetracycline were acquired from Sigma‐Aldrich (St. Louis, MO, USA), methylparaben was acquired from Acros (Geel, Belgium), and a formulation similar to the phytofungicide ARAW™ was prepared in our laboratory using the manufacturer's technical sheet (3.2% p/p eugenol; 6.4% p/p geraniol; 6.4% p/p thymol).^16^

#### 2.3.2 Standards sample preparation and GC conditions

##### 2.3.2.1 GC–FID conditions

A fraction of each EO (20 mg) was dissolved in 1 mL of *n*‐hexane, filtered, and injected into the GC–FID system. A gradient concentration calibration curve was prepared for each standard (0.25–10 mg/mL).

The EO was quantified using an Agilent 7890A gas chromatograph (Agilent, Santa Clara, CA, USA) equipped with an Agilent 7693 autosampler. A Supelco SPB™PUFA fused silica capillary column (300 × 0.25 mm i.d.) with a 0.20‐μm film was used (Supelco, Bellefonte, CA, USA), with helium as the carrier gas at a flow rate of 800 μL/min. A gradient temperature program was used, with the GC oven temperature starting at 60 °C for 2 min, increasing to 220 °C at 2 °C/min, and holding for 20 min, for a total runtime of 102 min. The injector temperature was set to 250 °C with a split ratio of 60:1. The sample injection volume was 1 μL, and a delay time of 4 min was applied to minimize interference with the solvent elution. The FID detector was set at 250 °C with air and hydrogen at flow rates of 400 and 40 mL/min, respectively. Data analysis was performed using proprietary software A.01.04. All assays were conducted in triplicate to guarantee statistical significance. The data were analyzed using one‐way analysis of variance (ANOVA) followed by Tukey's multiple comparison test using IBM SPSS Statistic 29.0.1.0 software (IBM Corp, Armonk, NY, USA) and principal component analysis of the identified peaks using MetaboAnalyst 6.0 software (Alberta, Canada).

Method validation, described in Table 2, was assessed in terms of linearity and sensitivity [limit of detection (LOD) and limit of quantification (LOQ)]. Linearity was measured using standard calibration curves that were fitted using least‐squares linear regression. The linearity was determined by determining the individual standard concentration and plotting the relative area *versus* concentration. The LOD and LOQ were determined by multiplying 3 and 10, respectively, by the ratios of the standard deviation(s) (SD) of the calibration curve interception to the regression slope.

**Table 2:** Parameters of method validation for gas chromatography–flame ionization detection quantification

ID	RT (min)	*R* ^2^	Range (mg/mL)	Equation	LOD (mg/mL)	LOQ (mg/mL)
(−) *α*‐Pinene	4.87	0.999	10–0.25	*y* = 282.6*x* + 30.504	0.16	0.54
(−) *β*‐Pinene	6.54	0.998	10–0.25	*y* = 285.1*x* + 54.556	0.49	1.64
(+)‐*p*‐Menth‐1‐ene	7.55	0.995	10–0.25	*y* = 216.9*x* + 6.836	1.06	3.54
*α*‐Terpinene	8.66	0.995	10–0.25	*y* = 104.58*x* + 20.177	0.74	2.45
*R* (+)‐Limonene	9.35	0.999	10–0.25	*y* = 301.96*x* − 45.859	0.45	1.51
*γ*‐Terpinene	11.12	0.994	10–0.25	*y* = 140.51*x* − 7.348	0.92	3.06
*p*‐Cymene	11.82	0.999	10–0.25	*y* = 165.85*x* + 21.395	0.35	1.17
(+/−)‐Citronellal	23.34	0.998	10–0.25	*y* = 175.98*x* + 16.087	0.51	1.71
(−)‐Isopulegone	27.24	0.990	10–0.25	*y* = 182.83*x* + 57.183	0.76	2.54
(−)‐Pulegone	28.17	0.996	10–0.25	*y* = 204.18*x* + 15.268	0,96	3.19
Carvacrol methyl ether	29.44	0.999	10–0.25	*y* = 181.22*x* − 0.186	0.40	1.35
(−)‐*β*‐Caryophyllene	30,49	0.999	10–0.25	*y* = 211.85*x* − 1.8877	0.25	0.82
(−)‐menthol	31.33	0.996	10–0.25	*y* = 124.91*x* + 13.355	0.77	2.57
*Trans*‐cinnamaldehyde	51.10	0.979	10–0.25	*y* = 114.53 *x* + 83.861	1.83	6.12
Eugenol	57.60	0.997	10–0.25	*y* = 288.88*x* + 4.966	0.41	1.38
Thymol	59.49	0.998	10–0.25	*y* = 282.11*x* − 65.011	0.39	1.29
Carvacrol	60.72	0.999	10–0.25	*y* = 304.22*x* + 6.852	0.23	0.76

LOD, limit of detection; LOQ, limit of quantification; *R* ^2^, correlation coefficient; RT, retention time.

##### 2.3.2.2 Gas chromatography–mass spectrometry conditions

For EO identification, a fraction of each EO (10 mg) was dissolved in methanol at a 1:50 proportion, filtered, and injected into the GC–MS. Separation and qualitative analysis were performed using an Agilent 6890 N gas chromatograph equipped with an Agilent 5975 Inert Mass Selective Detector. An Agilent J&W HP‐5 (5%‐phenyl)‐methylpolysiloxane nonpolar column (300 × 0.32 mm i.d.) with a 0.25‐μm film thickness was used. The oven temperature profile described for GC–FID was used, with a constant helium column flow of 1.0 mL/min. As for mass spectrometry (MS) conditions, the operating temperatures of the transfer line, quadrupole and ionization source were 250, 150 and 230 °C, respectively. Electro impact mass spectra were recorded at 70 eV, with an ionization current of 10 μL, and data acquisition was performed in scan mode (30–200 *m*/*z*). Identification of chromatographic peaks was performed by reference to data system library NIST Mass Spectral Search Program v 2.2–2005 software (Institute of Standards and Technology, Gaithersburg, MD, USA). To obtain the reference retention indices for the identified compounds and allow their comparison with retention indices described in the literature for similar experimental conditions,^17^, ^18^ the Supelco C7‐C40 Saturated Alkanes Mix Standard (St. Louis, MO, USA) was analyzed under the same experimental conditions.

### 2.4 Fungicide and fungistatic activity

#### 2.4.1 Phytopathogenic fungi

In this study, colonies of *A. alternata*, *B. cinerea*, and *F. oxysporum* were obtained from the Colección Española de Cultivos Tipo and maintained under laboratory conditions on PGA (Sigma‐Aldrich) at 25 ± 1 °C under darkness. To obtain a large population of pure colonies, a 7‐day colony‐repicking period on a fresh medium was established.

Antifungal activity was assessed using the agar dilution methodology^19^ with somemodifications.^20^, ^21^ The culture medium consisted of PGA at 39 g/L with the addition of the broad‐spectrum antibiotic tetracycline (Sigma‐Aldrich) at 10 μg/mL. For each EO, a stock solution of 50 mg/mL in 96% ethanol was prepared and added to 4.9 mL aliquots of molten culture medium at five different concentrations (0.01, 0.05, 0.1, 0.5, and 1 mg/mL). The solution was poured into a sterile Petri dish. Blank culture medium was used to prepare the negative control, and the commercial fungicide methylparaben (methyl‐4‐hydrobenzoate) was used as a positive control, with the same concentration gradient. On each plate, disks of the inoculum were removed from 7‐day colonies of each phytopathogenic fungus and placed at eight equidistant points, with each point as a replica. The dishes were incubated for 48 h (*B. cinerea*) or 72 h (*F. oxysporum* and *A. alternata*) at 25 ± 1 °C under darkness. For the evaluation of antifungal activity, the growth or inhibition of colonies was monitored by digitalizing the plates and determining the diameter of the treated colonies with respect to the controls using the image processing software ImageJ 1.43 (ImageJ, Bethesda, MD, USA). Mycelial growth inhibition was analyzed by ANOVA, and the half‐maximal inhibitory concentration (IC_50_) was estimated via a nonlinear regression using a sigmoidal dose‐response curve with normalized data with GraphPad Prism 10 (GraphPad Software, Boston, MA, USA).

#### 2.4.2 Obligate biotrophic fungi

Owing to the life cycle and pathogenicity of biotrophic fungi, such as those of the Erysiphaceae family associated with powdery mildew disease, antifungal activities were assessed using the detached leaf method. This qualitative technique allows screening of antifungal activity by evaluating mycelial growth or inhibition. Young leaves from the apple orchard *Malus domestica* (Suckow) Borkh. with initial symptoms of *Oidium farinosum* were collected and placed in a Petri dish, one per dish, with enough water to maintain the humidity between 80% and 100%. For each EO, 400 μL of solution at six different concentrations in 96% ethanol (2.5, 5.0, 7.5, 10.0, 15.0 and 20.0 μL/mL) was assessed. The solvent was used as the negative control. The commercial fungicide methylparaben (methyl‐4‐hydrobenzoate) and homemade phytofungicide ARAW™ were selected as positive controls, with the same concentration gradient. The assay had a maximum duration of 14 days, and the mycelial area on the leaf was measured after 14 days of incubation. Mycelial growth for each EO was normalized by measuring the foliar areas.

## 3 RESULTS AND DISCUSSION

### 3.1 Phytochemical analysis

**Figure 1:**
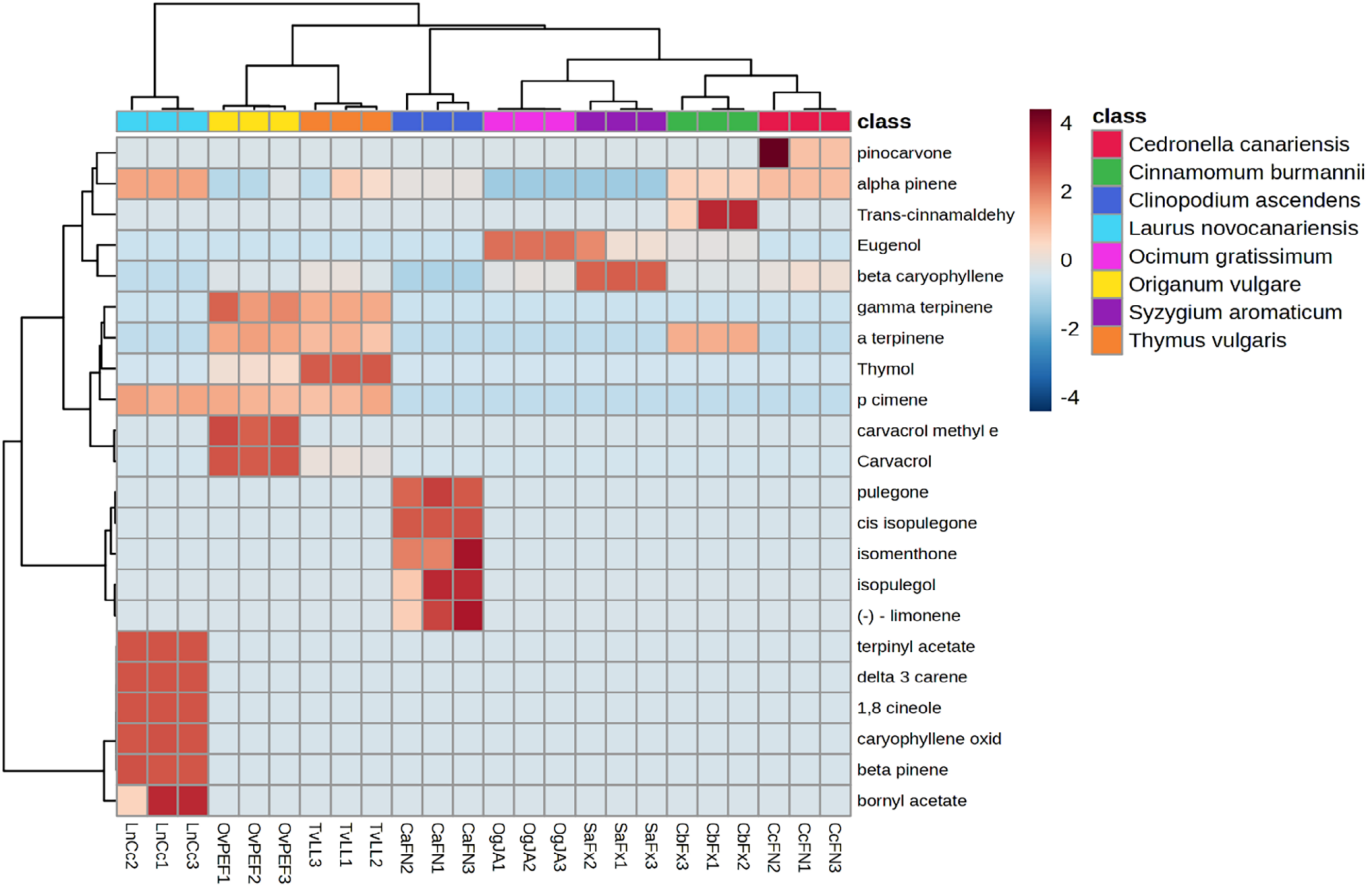
Hierarchical clustering heatmap for the essential oil profile.

Table 3 presents the principal chemical composition of each EO, including the retention indices (RI) determined using both a polar column (GC–FID) and a nonpolar column (GC–MS), along with the relative percentages of the major constituents. RI values were assigned based on comparisons with a retention index database and literature. Notably, significant RI variations exceeding 10 index units (IU) were observed for several compounds on the polar column. This discrepancy, previously described by Babushok *et al*.^18^ and supported by the retention indexes in the NIST Mass Spectrometry Data Center,^17^ could be attributed to the slight differences in the polarities of the polyethylene glycol stationary phases. Figure 1 shows the hierarchical clustering heatmap for the relationship between EOs based on the relative proportion of their major constituents.

**Table 3:** Essential oil profile characterization by gas chromatography with a flame ionization detector and by mass spectrometry

Chemical class	ID	RI^†^	RI^‡^	*Cedronella canariensis*	*Cinnamomum burmannii*	*Clinopodium ascendens*	*Laurus novocanariensis*	*Ocimum gratissimum*	*Origanum vulgare* subsp. *virens*	*Syzygium aromaticum*	*Thymus vulgaris*
				Relative % ± SD	Relative % ± SD	Relative % ± SD	Relative % ± SD	Relative % ± SD	Relative % ± SD	Relative % ± SD	Relative % ± SD
Monoterpene and monoterpenoid	*α‐*Pinene	1011	910	1.137 ± 0.14^a^	0.749 ± 0.01	0.275 ± 0.01	1.768 ± 0.03	—	0.403 ± 0.06	—	0.701 ± 0.16
	*β*‐Pinene	1108	966	—	—	—	0.535 ± 0.05	—	—	—	—
	*Δ* ^3^‐carene	1110	1010	—	—	—	3.562 ± 0.02^a,b^	—	—	—	—
	*p*‐Menthan‐3‐one	1272	1177	—	—	1.184 ± 0.304	—	—	—	—	—
	*α*‐Terpinene	1168	1020	—	0.715 ± 0.01	—	—	—	0.772 ± 0.06	—	0.611 ± 0.09
	Limonene	1207	1022	—	—	2.317 ± 0.55	—	—	—	—	—
	1,8‐Cineole	1188	1025	—	—	—	5.018 ± 0.03^a^	—	—	—	—
	*γ*‐Terpinene	1245	1043	—	—	—	—	—	5.968 ± 0.38^a,b^	—	3.267 ± 0.07
	*p*‐Cymene	1272	1024	—	—	—	2.177 ± 0.02	—	2.112 ± 0.29^a,b^	—	1.811 ± 0.29^a^
	Terpinen‐4‐ol	1598	1173	—	—	—	2.364 ± 0.19	—	—	—	—
	Pinocarvone	1571	1194	92.400 ± 1.67^a^	—	—	—	—	—	—	—
	Isopulegone	1513	1138	—	—	48.707 ± 1.75^a.b^	—	—	—	—	—
	Pulegone	1630	1220	—	—	32.331 ± 1.70	—	—	—	—	—
	Isopulegol	1660	1140	—	—	13.987 ± 1.99^a,b^	—	—	—	—	—
	Bornyl acetate	1577	1282	—	—	—	23.719 ± 0.02	—	—	—	—
	*α*‐Terpinyl acetate	1668	1329	—	—	—	15.644 ± 0.04 ^a,b^	—	—	—	—
	Thymol	2120	1288	—	—	—	—	—	5.650 ± 0.47^a^	—	89.495 ± 1.31^a,b^
	Carvacrol	2125	1290	—	—	—	—	—	73.036 ± 1.07^a,b^	—	1.360 ± 0.29^a^
Methyl ether terpene	Carvacrol methyl ether	1549	1231	—	—	—	—	—	1.961 ± 0.09	—	—
Sesquiterpene	*β*‐Caryophyllene	1601	1439	2.273 ± 0.40	0.883 ± 0.02	—	1.601 ± 0.03	1.110 ± 0.26	0.782 ± 0.05^a,b^	29.681 ± 0.95^a,b^	1.246 ± 0.453^a^
Sesquiterpene epoxide	Caryophyllene oxide	2090	1607	—	—	—	2.789 ± 0.04	—	—	—	—
Phenylpropanoid	*Trans*‐cinnamaldehyde	2025	1252	—	90.459 ± 3.97^a,b^	—	—	—	—	—	—
	Eugenol	2176	1338	—	—	—	—	94.555 ± 1.02^a,b^	—	67.477 ± 1.18^a,b^	—

*Note*: Mean of three replicates.

^a,b^Statistically significant differences according to one‐way ANOVA, followed by Tukey's test, *P* < 0.05.

^†^ Linear retention indices relative to C7–C40 *n*‐alkanes on a SPB PUFA polar column.

^‡^ Linear retention indices relative to C7–C40 *n*‐alkanes on a HP‐5 nonpolar column.

The most abundant volatile components identified ranged from two compounds in *S. aromaticum* to ten in *L. novocanariensis*. Chromatographic characterization revealed a predominance of monoterpenoids (27–92%) and monoterpenes (1–52%), with the sesquiterpene *β*‐caryophyllene being the most abundant in the majority of the studied EOs.

*Cedronella canariensis* exhibited a high content of the bicyclic ketone pinocarvone (92%); also present is *β*‐caryophyllene (2%) and traces of other monoterpenes. Previous studies^22^, ^23^ of this plant have shown pinocarvone levels below 70%, in most cases accompanied by the presence of its precursors *β*‐pinene, pinocamphone and isopinocamphone, which are absent in the current study. Seasonal variation is responsible for these differences, as previous experiments by our group, using plant material collected from the same location at a different time of year, found significantly lower pinocarvone levels—approximately 64% (unpublished data). Nevertheless, pinocarvone has been described as part of the OEs of many plants, comprising 20–30% in *Hyssopus officinalis* L.^24^ and 22% in *Adenophyllum porophyllum*,^25^ but always in much lower proportion than in *Cedronella*.

The *Clinopodium ascendens* EO was rich in pulegone (32%), its intermediate *cis*‐isopulegone (49%) and isopulegol (14%). This *cis*‐isopulegone chemotype was described by Castilho *et al*.^14^ for specimens collected from the same location and under similar conditions. Another study of wild specimens collected in Andalucía, Spain, revealed some differences in EO composition, with isomenthone (37%), pulegone (17%), and 1,8‐cineole (18%) being the prevalent compounds.^26^ Additionally, Eastern European samples contained high proportions of *cis*‐piperitone oxide (46%) and *cis*‐piperitone (66–75%), indicating distinct chemotypes variations.^27^, ^28^ These findings suggest the existence of two biosynthetic pathways: one derived from the isopiperitenone (common precursor) isomerase leading to piperitenone, piperitone, and their oxides, and the other from the isopiperitenone reductase leading to isopulegone and from there to pulegone and menthones. A similar observation was reported for *Calamintha nepeta* from Italy and Portugal, with the Italian plant showing high amounts of components from the isomerase and the Portuguese plant showing products of the isopiperitenone reductase.^29^ Isopulegone isomerase (converting isopulegone to pulegone), identified in 2018, is considered the ‘missing link’ in the menthol biosynthetic pathway and exhibits environmental sensitivity.^30^ Plants from the same location, collected at separate times of the year, show remarkable variations on the ratio isopulegone/pulegone + menthone. Moreover, plants obtained by micropropagation of the wild specimens and cultivated under controlled irrigation and fertilization show a quite different ratio of these ketones when compared to the wild plant.

The composition of the leaf oil of *L. novocanariensis*, apart from not showing no relationship with the chromatographic profiles of the other studied EOs, differs significantly from the other species of *Laurus* (*nobilis* and *azorica*), where 1,8‐cineole is usually dominant. Although 1,8‐cineole was present, the acetate ester monoterpenoid fraction was more prominent. In our EO, 1,8‐cineole accounted for approximately 5%, whereas bornyl acetate represented 24% and *α*‐terpinyl acetate was about 16%. The sesquiterpenoid fraction contained *β*‐caryophyllene (2%) and caryophyllene oxide (3%), along with numerous unidentified sesquiterpenoid alcohols and esters, each comprising less than 0.5% of the EO weight. Previous studies have suggested the presence of different *L. novocanariensis* chemotypes on Madeira Island, which is rather unlikely.^31^ Unpublished results from our research group using leaves picked from the same specimen revealed remarkable variation between young and mature leaves, male and female tree leaves, and variations throughout the year. The variability is remarkable in the more polar compounds: the 40% ‘other’ components mentioned in Table 3 correspond to a numerous group of sesquiterpenoids, mainly alcohols and esters, with lots of isomers.

For *Cinnamomum burmannii*, *Ocimum gratissimum*, and *S. aromaticum*, phenylpropanoids composed most of the volatiles, with *trans*‐cinnamaldehyde being the most abundant component (90%) in *Cinnamomum burmannii* and eugenol being the most prevalent in *Ocimum gratissimum* (95%) and *S. aromaticum* (68%). Analysis of the heatmap (Fig. 1) reveals that *Ocimum gratissimum* and S. *aromaticum* share similar chromatographic profiles, forming a distinct cluster, while *Cinnamomum burmannii* is more similar to *Cedronella canariensis*, due to the presence and relative abundance of α‐pinene. Wang *et al*.^32^ proposed that *Cinnamomum burmannii* is the most common source of cinnamon in Europe and United States. Our results confirm this conclusion, as no eugenol was present in *our* cinnamon sample, implying a positive identification of the species as *Cinnamomum burmannii*. Other volatiles in cinnamon accounted for less than 10%.

The results for *Ocimum gratissimum* were similar to those of Oliveira *et al*.^33^ for the same species, with eugenol (90%) as the major compound. The volatile composition is remarkably similar to *S. aromaticum*, with 67% of the EO identified as eugenol and 30% as *β*‐caryophyllene. The prevalence of eugenol has been described in the literature,^34^, ^35^ although the proportion of *β*‐caryophyllene was higher in our study.

Carvacrol and its stereoisomer thymol are among the most abundant components of *Origanum vulgare* subsp. *virens* and *T*. *vulgaris*, respectively. Both stereoisomers are present in these EOs, along with their intermediates *γ*‐terpinene and *p‐*cymene. Owing to the similarities in their chromatographic profiles, these two EOs were grouped into the same cluster. Krause *et al*.^36^ identified the biosynthetic pathway for thymol and carvacrol, with the precursor γ‐terpinene oxidized by cytochrome P450 monooxygenases from the CYP71D family, leading to the formation of unstable cyclohexadienol intermediates. These intermediates are then dehydrogenated by the short‐chain dehydrogenase/reductase TvSDR1 to produce ketone intermediates, which form the aromatic backbone of thymol and carvacrol via keto‐enol tautomerism, respectively. In our study, the *Origanum vulgare* EO sample was found to be characteristic of a carvacrol chemotype.

### 3.2 Fungicide and fungistatic activity

#### 3.2.1 Phytopathogenic fungi

**Figure 2:**
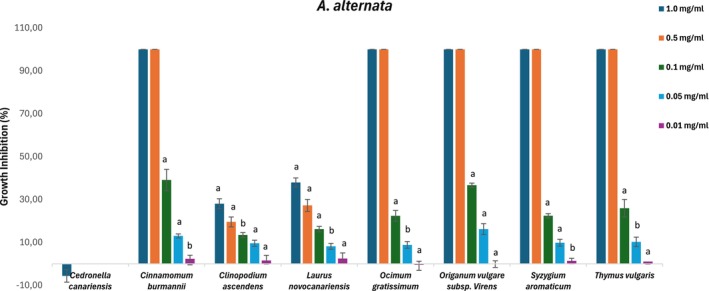
Antifungal effects (percentage growth inhibition) of the essential oils against *Alternaria alternata*. Subfractions with a growth inhibition greater than 20% at 1 mg/mL were assayed at lower concentrations (0.5, 0.1, 0.05 and 0.01 mg/mL). The nonactive fractions have been omitted. The results are expressed as a percentage relative to the negative control. The data are presented as the mean ± SD (*n* = 8). a, b: significant differences determined by one‐way ANOVA, *P* < 0.05.

**Figure 3:**
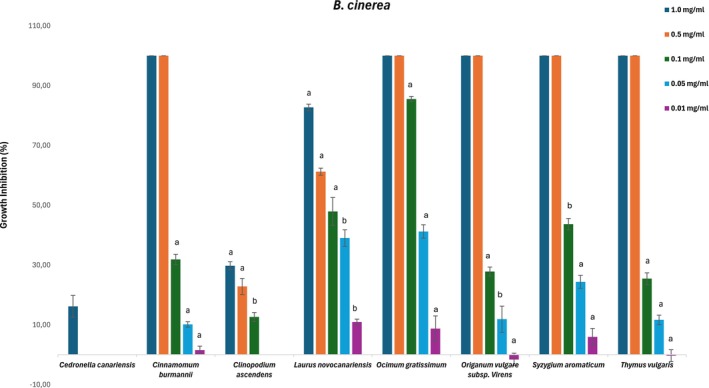
Antifungal effects (percentage growth inhibition) of the essential oils against *Botrytis cinerea*. Subfractions with a growth inhibition greater than 20% at 1 mg/mL were assayed at lower concentrations (0.5, 0.1, 0.05 and 0.01 mg/mL). The nonactive fractions have been omitted. The results are expressed as a percentage relative to the negative control. The data are presented as the mean ± SD (*n* = 8). a, b: significant differences determined by one‐way ANOVA, *P* < 0.05.

**Figure 4:**
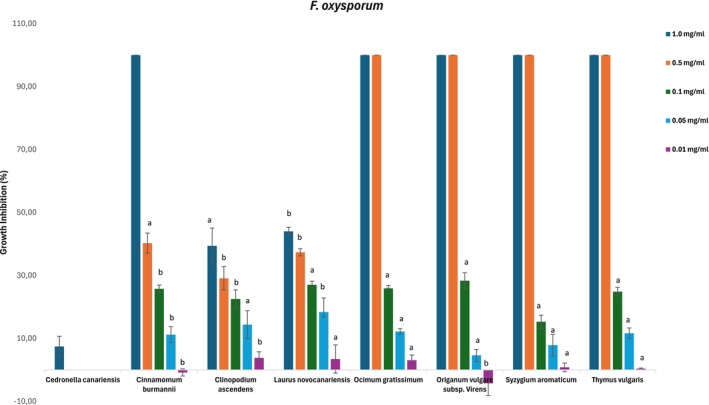
Antifungal effects (percentage growth inhibition) of the essential oils against *Fusarium oxysporum*. Subfractions with a growth inhibition greater than 20% at 1 mg/mL were assayed at lower concentrations (0.5, 0.1, 0.05 and 0.01 mg/mL). The nonactive fractions have been omitted. The results are expressed as a percentage relative to the negative control. The data are presented as the mean ± SD (*n* = 8); a, b: significant differences determined by one‐way ANOVA, *P* < 0.05.

The selected EOs were evaluated against *B. cinerea*, *F. oxysporum*, and *A. alternata* to determine the growth inhibition thresholds and inhibitory concentrations (IC_50_). Figures 2, 3 and 4 represent the perceived antifungal effects (% growth inhibition) of the EOs against *A. alternata*, *B. cinerea*, and *F. oxysporum*, with subfractions showing a growth inhibition greater than 20% at 1 mg/mL assayed at lower concentrations (0.5, 0.1, 0.05, and 0.01 mg/mL).

According to Table 4 and Figs 2, 3 and 4, susceptibility varied among the three fungal species, with *B. cinerea* displaying weaker resistance than *A. alternata* and *F. oxysporum* for most of the tested EOs in this study. The inhibition of growth by *Cedronella canariensis* at 1.0 mg/mL was almost negligible for *F. oxysporum* (7%), and the growth area was greater than that of the control solvent for *A. alternata* (6%); therefore, this EO was not screened at lower concentrations. López‐Garcia *et al*.^37^ determined the qualitative and quantitative compositions of EOs from the aerial parts of two varieties of *Cedronella canariensis* and observed high antifungal activity against *Candida* spp. and *Saccharomyces cerevisiae*, albeit at higher concentrations than those used in this study. However, these results have not been confirmed.

**Table 4:** Inhibition concentration (IC_50_, mg/mL) and gradient concentration of each essential oil for *Botrytis cinerea*, *Alternaria alternata* and *Fusarium oxysporum*

Essential oils	Inhibition concentration IC_50_ (mg/mL) and gradient concentration *t* = 48^†^ and 72 h								
	*B. cinerea*			*A. alternata*			*F. oxysporum*		
	IC_50_	Pearson correlation coefficient r *P*‐value	Gradient concentration *R* ^2^	IC_50_	Pearson correlation coefficient r *P*‐value	Gradient concentration *R* ^2^	IC_50_	Pearson correlation coefficient r *P*‐value	Gradient concentration *R* ^2^
*Cedronella canariensis*	4.451*	0.9837 0.0025	n.d.	n.d.	0.9766 0.0043	n.d.	7.937*	0.9956 0.0004	n.a.
*Cinnamomum burmannii*	0.131 (0.12–0.14)	0.9502 0.0132	*y* = −28.685*x* + 134.750 0.888	0.118 (0.11–0.12)	0.9601 0.0095	*y* = −28.230*x* + 135.570 0.910	0.343 (0.26–0.63)	0.8885 0.0440	*y* = −23.070*x* + 104.480 0.8601
*Clinopodium ascendens*	6425* (4.58–9.95)	0.9933 0.0736	*y* = −8.580*x* + 38.877* *0*.988	8.078* (5.74–12.26)	0.9855 0.0021	*y =* −6.292*x* + 33.304 0.986	2763* (1.95–4.37)	0.9869 0.0018	*y =* −8.595*x* + 47.627 0.994
*Laurus novocanariensis*	0.137 (0.117–0.156)	0.9837 0.0025	*y* = −16.581*x* + 98.091 0.970	2.386* (2.04–2.86)	0.9766 0.0043	*y* = −9.005*x* + 45.421 0.984	2.243* (2.03–2.54)	0.9956 0.0004	*y =* −10.010*x* + 56.074 0.981
*Ocimum gratissimum*	0.056 (0.05–0.06)	0.9427 0.0163	*y* = −24.141*x* + 139.500 0.885	0.148 (0.14–0.16)	0.9389 0.0179	*y* = −29.211*x* + 133.780 0.859	0.143 (0.13–0.15)	0.9404 0.0173	*y* = −28.168*x* + 132.730 0.8625
*Origanum vulgare* subsp. *virens*	0.138 (0.13–0.15)	0,9536 0.0119	*y* = −29.135*x* + 135.020 0.886	0.121 (0.11–0.13)	0.9667 0.0073	*y* = −28.376*x* + 135.700 0913	0.131 (0.12–0.14)	0.9504 0.0132	*y* = −30.393*x +* 136.910 0.890
*Syzygium aromaticum*	0.105 (0.10–0.11)	0.9738 0.0051	*y* = −26.376*x* + 133.920 0.925	0.149 (0.14–0.16)	0.9357 0.0194	*y* = −28.740*x* + 132.950 0.853	0.162 (0.15–0.17)	0.9208 0.0265	*y* = −29.053*x* + 131.950 0.822
*Thymus vulgaris*	0.144 (0.13–0.15)	0.9469 0.014	*y* = −28.901*x* + 134.050 0.873	0.143 (0.13–0.15)	0.9430 0.016	*y* = −28.764*x* + 133.740 0.869	0.145 (0.14–0.15)	0.9443 0.0156	*y* = −28.757*x* + 133.650 0.868
Control (+)Methyl‐4‐hydrobenzoate	0.119 (0.11–0.13)	0.9613 0.009	*y* = −26.375*x +* 133.92 0.925	0.192 (0.18–0.20)	0.9444 0.016	*y* = −26.121*x* + 122.66 0.894	0.124 (0.11–0.14)	0.9873 0.002	*y* = −23.612*x* + 121.94 0.986

*P* < 0.05; IC_50_, concentration of the extract that induces 50% mycelial inhibition (95% confidence interval, bottom and upper limits).

* Best fit values for the dose–response curve.

^†^ Indicates incubation time for *B*. *cinerea*.

n.d., not determined.

The high content of *trans*‐cinnamaldehyde described for *Cinnamomum burmannii* likely contributes to the IC_50_ values observed for *B. cinerea* and *A. alternata* (Table 4, Figs 2 and 3); this was the most effective EOs for inhibiting *A. alternata* and *F. oxysporum*, with slight weaker activity against the later. The antifungal properties of this phenylpropanoid have been previously described.^38^, ^39^, ^40^ Wang *et al*.^41^ described the antifungal mode of action of cinnamaldehyde by inhibiting spore germination and hyphal growth and modulating the oxidative stress response, leading to the inhibition of aflatoxin B1 biosynthesis.

*Clinopodium ascendens* and *L. novocanariensis* presented weak to moderate antifungal activity. For *Clinopodium ascendens*, the inhibition growth rate at 1.0 mg/mL was less than 50% for the three fungi, and the IC_50_ values were above 1.0 mg/mL. The relative proportions of secondary metabolites, notably pulegone and *cis*‐isopulegone, appear to reflect the weak activity of the studied fungi, as mentioned by Castilho *et al*.,^14^ who described a 20% growth inhibition rate against *B. cinerea*. Another study by Montenegro *et al*.^42^ evaluated the antifungal activity of pulegone, menthone, and isopulegol, among other volatiles, with pulegone IC_50_ values of 0.49 mg/mL for *B. cinerea* and 0.69 mg/mL for *Monilinia fruticola*. Another study by Griffin *et al*.^19^ demonstrated that isopulegol and menthol have higher activity than the ketones menthone and pulegone.

For *L. novocanariensis*, mycelial growth inhibition was more visible for *B. cinerea*, with an 83% inhibition for the upper tested concentrations and <50% for the other fungi (Fig. 3). Yong *et al*.^43^ evaluated the antifungal activity against *B. cinerea* for EO extracted from the leaves of three conifer species and observed highest antifungal activity for the species in which bornyl acetate and terpinyl acetate were predominant and no difference in antifungal activity between the EO treatment and the two monoterpene ester treatment. Another study by Kusumoto *et al*.^44^ described the higher mycelium inhibition for terpinyl acetate and bornyl acetate in comparison with monoterpene hydrocarbons such as *α*‐pinene. Also, the monoterpenoid 1,8‐cineole, also present in relative proportion on *L. novocanariensis*, is known for its antifungal activity. A study by Shukla *et al*.^45^ described complete growth inhibition for *F. oxysporum* at 0.61 mg/mL. Morcia *et al*.^46^ concluded that 1,8‐cineole is active only at the highest concentrations (>0.426 mL per 100 mL of medium) over *Fusarium* spp. and *A. alternata*. It is thus hypothesized that the sesquiterpene and sesquiterpenoid fractions of *L. novocanariensis* containing *β‐*caryophyllene, the epoxy caryophyllene oxide, and several non‐identified alcohols with germacrene and eudesmane skeleton, could play a major role in antifungal activity.^47^

*Ocimum gratissimum* was the most effective at inhibiting *B. cinerea* mycelial growth, with an IC_50_ value of 0.05 mg/mL. *S. aromaticum* also showed complete inhibition at concentrations ≥0.5 mg/mL, highlighting the antifungal activity of the phenylpropanoid eugenol, the predominant component of these two EOs (Figs 2, 3 and 4). Various studies^48^, ^49^, ^50^ have described the correlation between the antifungal mechanism of action and the high content of eugenol in EOs, suggesting that eugenol exerts its activity through direct damage to the fungal cell membrane and by impairing the biosynthesis of ergosterol, an essential structural component of fungal cells.

The growth inhibition rates of *Origanum vulgare* subsp. *virens* and *T. vulgaris* were quite similar for the three phytopathogenic fungi; *Origanum* was more efficient for *A. alternata* (Table 4 and Figs 2, 3, 4). The antifungal activity of these EOs is due to the presence of high amounts of monoterpenoids, with an abundance of the isomer carvacrol (73%) occurring in *Origanum vulgare*, and thymol (89%) occurring in *T. vulgaris*. The antifungal properties of carvacrol‐ and thymol‐rich EOs are well described in the literature; for example, ethnopharmacological use of *Origanum vulgare* L. has been described on multiple academic platforms,^51^, ^52^, ^53^, ^54^, ^55^ and antifungal activity is related to the disruption of fungal cell wall integrity and the impairment of ergosterol synthesis.^56^

#### 3.2.2 Obligate biotrophic fungi

As shown in Table 5, the antifungal activity of the tested EOs against the obligate biotrophic fungus *Oidium farinosum* can be categorized into clusters based on their efficacy. In the negative control treatment (96% EtOH), an average of 79% of the upper leaf surface was covered by powdery mildew. Among the fungicides used as positive controls, commercial methyl paraben exhibited the highest efficacy, reducing surface infection from 31% to 18%, with its effectiveness increasing proportionally with concentration.

**Table 5:** Area of *Oidium farinosum* mycelium growth per leaf for each essential oil

Essential oil	Area of mycelium growth per leaf (%)					
	Gradient concentration (μl/mL)					
	2.5	5	7.5	10	15	20
*Cedronella canariensis*	86	76	81	81	78	82
*Cinnamomum burmannii*	78	45	34	28	24	22
*Clinopodium ascendens*	89	83	81	75	76	45
*Laurus novocanariensis*	78	82	82	79	84	83
*Ocimum gratissimum*	82	76	41	28	24	20
*Origanum vulgare*	83	79	82	37	28	21
*Syzygium aromaticum*	84	83	76	31	23	24
*Thymus vulgaris*	78	80	81	49	34	36
Control (+) Methyl‐4‐hydrobenzoate	31	26	28	22	28	18
Control (+) ARAW	41	42	38	33	31	32

Phenylpropanoid‐rich EOs, including *Ocimum gratissimum*, *S. aromaticum*, and particularly *Cinnamomum burmannii*, demonstrated the strongest antifungal activity, significantly reducing powdery mildew severity. At the highest tested concentration (20 μL/mL), these EOs decreased the diseased area to less than 25%, outperforming the commercial fungicide at 15 μL/mL. However, the volatile nature of these extracts may have limited their ability to sustain efficacy compared with the more stable commercial formulation. Notably, no mycelial growth inhibition was observed at the lowest tested concentrations, suggesting that effective inhibition requires concentrations above 7.5 μL/mL for *Cinnamomum burmannii* and 10.0 μL/mL for *Ocimum gratissimum* and *S. aromaticum*. The antifungal properties of *trans*‐cinnamaldehyde (*Cinnamomum burmannii*) and eugenol (*Ocimum gratissimum* and *S. aromaticum*) are further discussed in relation to their effects on phytopathogenic fungi.

A second cluster comprised the EOs of *Origanum vulgare* and *T. vulgaris*, which exhibited moderate antifungal activity. The presence of monoterpenoid isomers—carvacrol in *Origanum vulgare* and thymol in *T. vulgaris*—was associated with a reduction in mycelial growth at an intermediate concentration of 10 μL/mL. These findings align with previous reports by Mostafa *et al*.,^57^ which documented the efficacy of thyme oil against *Podosphaera xanthii*, the causative agent of cucumber powdery mildew.

Conversely, *Cedronella canariensis* and *L. novocanariensis* did not inhibit mycelial growth, whereas *Clinopodium ascendens* displayed antifungal activity only at the highest tested concentration. Despite the known antifungal properties of bornyl acetate and terpinyl acetate, which have been studied in relation to conidial germination inhibition^44^, ^45^, ^58^, the concentrations used in this assay may have been insufficient to elicit a detectable antifungal effect.

Donnarumma *et al*.^58^ evaluated the activity of EOs extracted from *Rosmarinus officinalis*, *S. aromaticum* and *Origanum vulgare* against powdery mildew growing on zucchini plants (*Cucurbita pepo* L.) and concluded that a formulation of these EOs were able to reduce disease incidence and severity more effectively than the individual EOs, thus demonstrating synergistic action.

## 4 CONCLUSION

This study demonstrates that the antifungal efficacy of EOs depends strongly on their major chemical constituents. Phenylpropanoid‐rich oils, particularly from *Cinnamomum burmannii*, *Ocimum gratissimum*, and *S. aromaticum*, were the most potent against phytopathogenic fungi and the obligate biotroph *Oidium farinosum*. Among terpenoid‐rich EOs, those dominated by thymol (*T. vulgaris*) and carvacrol (*Origanum vulgare* subsp. *virens*) exhibited antifungal effects, with carvacrol being slightly more effective across the tested phytopathogenic fungi. The observed differences suggest that quantitative variations in EO composition and isomerism influence antifungal activity. Overall, our findings support the general classification of EO antifungal activity based on major chemical constituents, as proposed by Kurita and Koike,^59^ adding phenylpropanoids as an even more efficient class: phenylpropanoids > phenols > alcohols > ketones > ethers > hydrocarbons.

Our results align with previous reports on EO efficacy against phytopathogenic fungi and support their potential application in sustainable pest management strategies. Notably, this study provides new insights into EO components rarely evaluated for antifungal activity, such as pinocarvone and isopulegone, which are typically minor constituents in EOs, but very abundant in some of the plants under study. In addition, the assessment of biological activities against obligate biotrophic fungus responsible for the powdery mildew represents a significant contribution, because this group of pathogens is often overlooked in biopesticide research.

However, further research is needed to elucidate the mechanisms of action of the primary EO constituents, as well as their synergistic or antagonistic interactions. In addition, future studies should explore their impacts on non‐target organisms and develop strategies for controlled release, degradation, and cost‐effectiveness to enhance their practical application as biopesticides.

## AUTHOR CONTRIBUTIONS

Conceptualization, RF and PC. Methodology, RF, DS, and SS. Investigation, RF and VP. Writing—original draft, RF and VP. Writing—review and editing, PC, DS, and RC. Funding acquisition, PC. All authors have read and agreed to the submitted version of the manuscript. The authors declare that they have no known competing financial interests or personal relationships that could have appeared to influence the work reported in this paper.

## Data Availability

The data that support the findings of this study are available from the corresponding author upon reasonable request.
